# Histone Methylation in Nickel-Smelting Industrial Workers

**DOI:** 10.1371/journal.pone.0140339

**Published:** 2015-10-16

**Authors:** Li Ma, Yana Bai, Hongquan Pu, Faxiang Gou, Min Dai, Hui Wang, Jie He, Tongzhang Zheng, Ning Cheng

**Affiliations:** 1 School of Public Health, Lanzhou University, Lanzhou, Gansu, P.R. China; 2 Workers’ Hospital of Jinchuan Company, Jinchuan Group CO., LTD, Jinchang, Gansu, P.R. China; 3 College of Basic Medicine, Lanzhou University, Lanzhou, Gansu, P.R. China; 4 Cancer Institute and Hospital, Chinese Academy of Medical Sciences, Beijing, China; 5 School of Public Health, Yale University, 60 College Street, New Haven, Connecticut, United States of America; New York University School of Medicine, UNITED STATES

## Abstract

**Background:**

Nickel is an essential trace metal naturally found in the environment. It is also common in occupational settings, where it associates with various levels of both occupational and nonoccupational exposure *In vitro* studies have shown that nickel exposure can lead to intracellular accumulation of Ni^2+^, which has been associated with global decreases in DNA methylation, increases in chromatin condensation, reductions in H3K9me2, and elevated levels of H3K4me3. Histone modifications play an important role in modulating chromatin structure and gene expression. For example, tri-methylation of histone H3k4 has been found to be associated with transcriptional activation, and tri-methylation of H3k27 has been found to be associated with transcriptional repression. Aberrant histone modifications have been found to be associated with various human diseases, including cancer. The purpose of this work was to identify biomarkers for populations with occupational nickel exposure and to examine the relationship between histone methylation and nickel exposure. This may provide a scientific indicator of early health impairment and facilitate exploration of the molecular mechanism underlying cancer pathogenesis.

**Methods:**

One hundred and forty subjects with occupational exposure to Ni and 140 referents were recruited. H3K4 and H3K27 trimethylation levels were measured in subjects’ blood cells.

**Results:**

H3K4me3 levels were found to be higher in nickel smelting workers (47.24±20.85) than in office workers (22.65±8.81; *P* = 0.000), while the opposite was found for levels of H3K27me3(nickel smelting workers, 13.88± 4.23; office workers, 20.67± 5.96; *P* = 0.000). H3K4me3 was positively (r = 0.267, *P* = 0.001) and H3K27 was negatively (r = -0.684, *P* = 0.000) associated with age and length of service in smelting workers.

**Conclusion:**

This study indicated that occupational exposure to Ni is associated with alterations in levels of histone modification.

## Introduction

Nickel is one of the most abundant trace metals on Earth, and is widely distributed in the general environment. It is used in various occupational settings, including in mining, smelting, and refining and in the alloy production and welding industries[1]. Workers employed in these industries are chronically exposed to nickel and related compounds, and non-occupational exposure is also common. Exposure may occur via inhalation of nickel-contaminated dust or air, ingestion of contaminated food or water, or other environmental sources such as tobacco and consumer products like stainless steel kitchen utensils and jewelry[2]. Existing epidemiological and experimental data have suggested that nickel, and other substances in the exposed environment can increase the risk of lung cancer and that nickel and various nickel compounds are carcinogens in humans[3,4]. Lung cancer is the leading cancer in China. Recent epidemiological investigations have revealed that occupational exposure to nickel is significantly closely associated with increased mortality of lung cancer among nickel smelting industry workers in China[5]. Over the past three decades, the morbidity and mortality associated with lung cancer have increased, bringing the overall 5-year survival rate down to below 15%, so better methods of early detection and treatment are needed[6].

Given that nickel and its compounds have been shown to have weak mutagenic capacity in bacterial and mammalian cell culture systems, the role of epigenetics has drawn attention as a direction to be explored[7]. Histone modifications play an important role in many nuclear processes, such as modulation of chromatin structure and gene transcription[8]. Tri-methylation of the histone H3k4 has been found to be associated with transcriptional activation, and tri-methylation of H3k27 has been found to be associated with transcriptional repression [9]. Aberrant histone modifications have been associated with various human diseases, including cancer[10]. *In vitro* studies have shown that nickel exposure can lead to intracellular accumulation of Ni^2+^, which is associated with global decreased DNA methylation, increased chromatin condensation, reduced H3K9me2, and elevated H3K4me3[11,12]. Most previous studies of histone modifications in subjects exposed to nickel have been case-control studies and animal experiments. Although there have been numerous cohort studies on nickel exposure, few have focused on changes in histone modifications. For this reason, the present cohort study focused on nickel-associated changes in global histone modifications using peripheral blood cells from nickel smelting factory workers in Jinchang, China. The purpose of this study was to identify biomarkers in populations with occupational nickel exposure, to determine the relationship between length of industrial service and nickel exposure, to provide a scientific indicator of early health impairment, and to explore the possible molecular mechanisms underlying the pathogenesis of cancer.

## Methods

### Ethics statement

Protocols involving this human cohort study were approved by the Ethics Committee of Lanzhou University. All participants provided written informed consent to their participation in this study.

### Subjects

Healthy subjects who had undergone a physical examination at the employee health center of Jinchuan Corporation or its secondary branches from June 1, 2011 to January 31, 2012 were enrolled in the present study (N = 12,388 subjects; 8,434 male).

To identify differences among nickel-smelting workers, subjects from secondary branches with the highest nickel exposure (nickel smelting factories), were included in the exposed group. Office workers were included in the control group.

### Exposed worker sampling

High-exposure positions in the company were identified using technical personnel familiar with the production process and the type and concentration of nickel compounds in the working environment. Among the 3,649 smelting workers, 969 had occupational exposure to high doses of nickel. The distribution of workers based on length of service (5-year increments) and age groups are shown in Table 1. To eliminate the confounding effects of age from the relationship between length of service and nickel exposure, a diagonal sampling method was used to select 20 subjects in each group for sampling (total subjects in exposed group = 140).

**Table 1:** Sampling distribution of high nickel exposure in groups with different lengths of service.

Years of Service	Age group (years)						
	20–24	25–29	30–34	35–39	40–44	45–49	50 and older
0–	20	—	—	—	—	—	—
5–	—	20	—	—	—	—	—
10–	—	—	20	—	—	—	—
15–	—	—	—	20	—	—	—
20–	—	—	—	—	20	—	—
25–	—	—	—	—	—	20	—
30+	—	—	—	—	—	—	20

### Control sampling

Office workers age-matched to subjects in the different exposure subgroups (within 3 years of difference) were selected. Seven age groups (5-year increments) with 20 subjects each were used (N = 140 control subjects).

### Division of job, occupational history, and calculation of service length

All workers enrolled in the initial survey completed a detailed occupational history with a chronological summary of work in any secondary branches of the Jinchuan Corp. All subjects selected for the exposure group had been employed in smelting factories as a first job and the length of service was defined as years worked at positions with exposure to high concentrations of nickel.

### Blood sample collection and handling

Fasting blood samples were collected in the morning and split into four aliquots with two anticoagulant and two nonanticoagulant collections (3 ml/each collection) for each recruited subject. After gently shaking, blood samples were centrifuged by a low-temperature ultracentrifuge at 2000 mp/min for 10 min at 4°C. Then 1 ml of serum or plasma was transferred to 1.8 ml frozen tubes and stored at -80°C. Blood samples were stored at 4°C and left at room temperature for 1 h before histone extraction using histone extraction kit (manufactured by Epigentek Group Inc. and the Cat. Log NO is OP-0006).

### Histone extraction

Histone extraction was performed according to the manufacturer’s instructions. Briefly, the histone extraction kit was left at room temperature to balance the temperature between the reagents and blood samples. Then 300 μl of serum or plasma was lysed with equal volume of cell lysis buffer from the kit. The lysate was gently mix for 10 min on ice followed by 10,000 rpm/min centrifugation at 4°C for 1 minute. The pellet was resuspended with 300 μl lysis buffer and incubated on ice for 30 min. This was followed by 12,000 rpm/min centrifugation at 4°C for 5 min. The supernatant was transferred into a new Eppendorf tube and diluted DTT buffer was added at a ratio of 0.3: 1 (0.3 ml diluted DTT in 1 ml supernatant). Samples were vortexed followed by 95°C incubation for 40 min. Samples were then cooled down with flowing water followed by 4000 rpm/min centrifuge for 10 min. The supernatant was measured for OD value at 532 nm with ddH2O as a blank control.

### Detection of global histone modification

Histone modifications were assessed using a sandwich enzyme-linked immunosorbent assay (ELISA).

### Statistical analysis

After natural logarithm conversion, the H3K27me3 and H3K4 me3 were found to fit normal distribution, So the data are here expressed as mean ± standard deviation ($\bar{x}$ ± ***s***). Differences in histone modifications in blood cells among groups were compared using two-sample Student’s t-test, Differences in histone modifications in blood cells between people with different lengths of service were compared using the F test.

## Results

### General information of the subjects from the nickel-smelting industry

In the experimental exposure group (nickel-smelting workers), 140 male subjects ranging in age from 20–55 years old (38.34±8.92) were recruited. In the control group (office workers), 140 male subjects ranging in age from 24–55 years old (38.34±8.89) were recruited. All subjects were healthy with no chronic diseases and no recent history of any medication.

### Analysis of the effects of time on tri-methylation of histone in the high-nickel-exposure group

**Fig 1:**
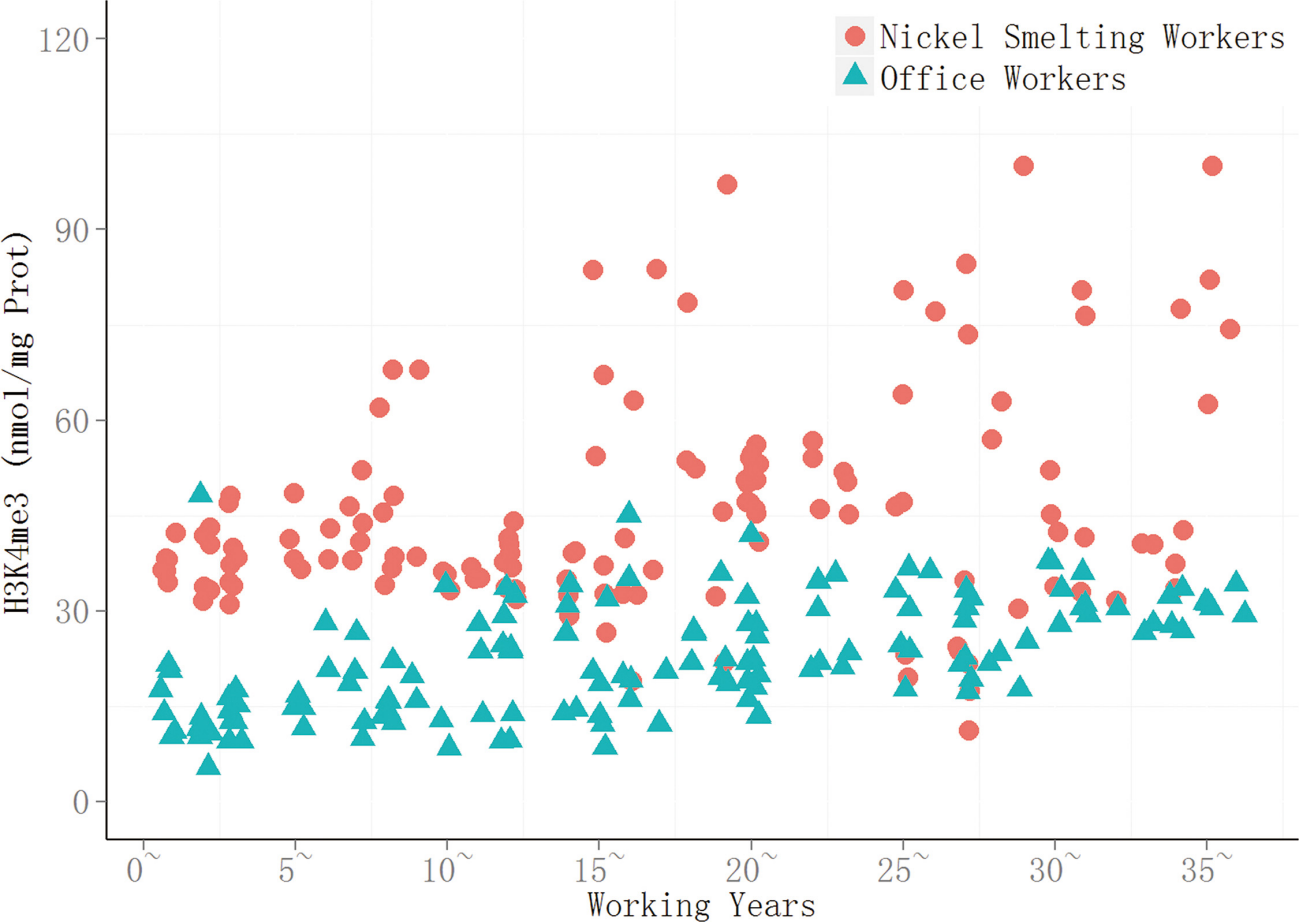
H3K4me3 in smelting and office workers with different working years. The level of H3k4me3 in every experimental subject, the red balls express the smelting workers, and green triangles express the office workers.

**Fig 2:**
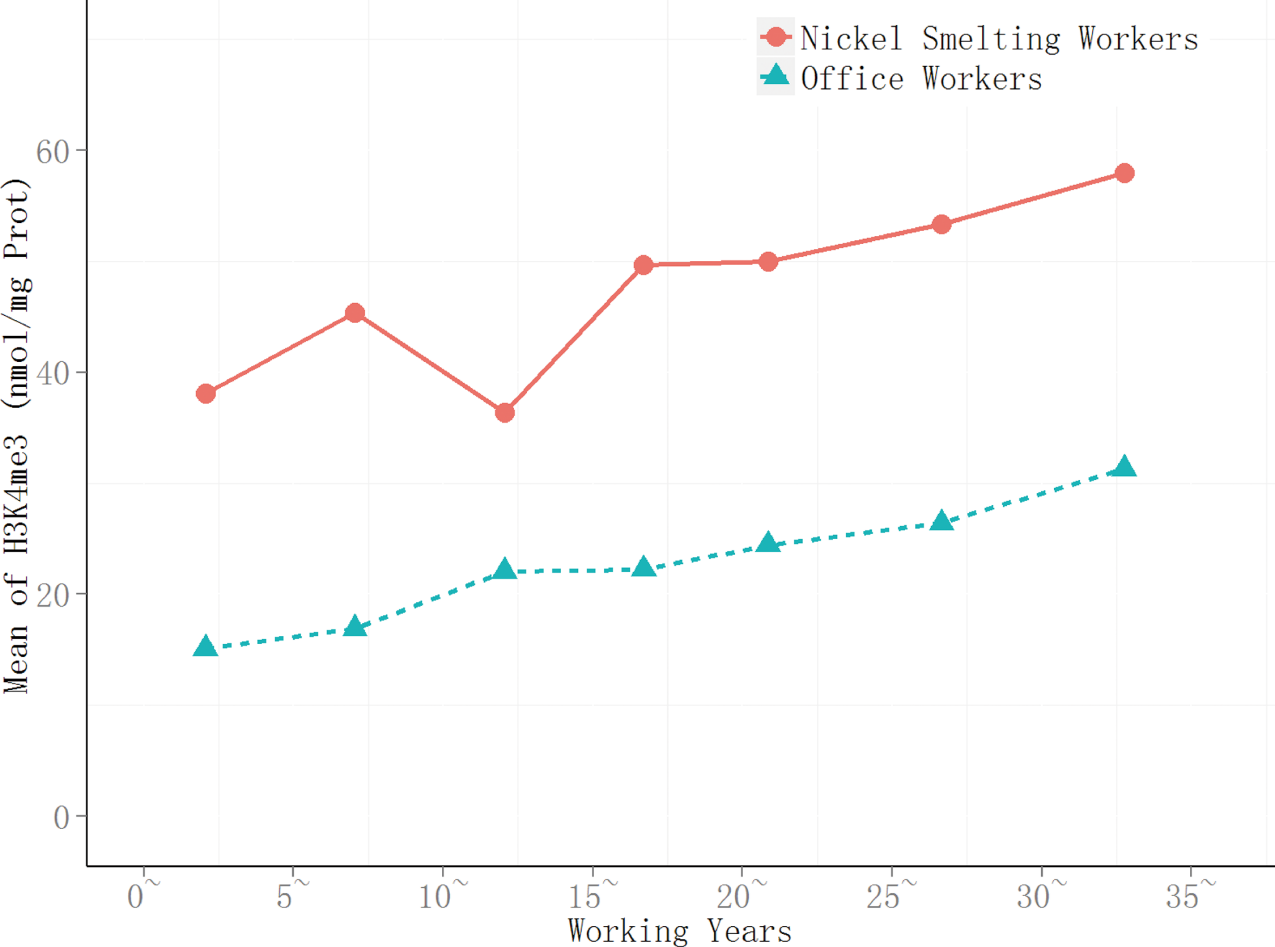
Trend of H3K4me3 with increasing working years. The trend of H3k4me3 in two group with incressing working years, the line connected with red balls express the smelting workers, and with green triangles express the office workers.

After natural logarithm conversion, the H3K4me3 data were found to fit normal distribution, there was heterogeneity of variance. For all length of service subgroups, t’ test indicated that there were significant differences in H3k4me3 level between the nickel-smelting workers and office workers (*P*<0.05) (Table 2).The level of H3k4me3 in subjects with high occupational exposure to nickel was significantly higher than in the control group (*P* < 0.05) (Figs 1 and 2). In the exposed group, the H3k4me3 levels in the 30+ service length subgroup were the highest,reaching a mean level of 58 nmol/mg prot. In the other service groups, H3k4me3 levels increased gradually with the length of service, and there was a significant positive correlation between the level of H3k4me3 and the length of service (r_s_ = 0.267, *P* = 0.001). In the control group, the level of H3k4me3 was also positively correlated with the length of service (r_s_ = 0.608,*P* = 0.000).

**Table 2:** H3K4me3 in nickel smelting and office workers with different lengths of service (nmol/mg prot).

Groups	working years (20 individuals per group)									
	0–4.99	5–9.99	10–14.99	15–19.99	20–24.99	25–19.99	30+	Total	r_s_	*P*
Smelting	37.71±1.13	44.26±1.22	36.23±1.11	45.15±1.58	49.90±1.09	42.52±2.01	52.98±1.52	47.24±20.85	0.267	0.001*
Office	13.60±1.54	16.28±1.31	20.09±1.60	20.70±1.51	23.34±1.36	25.79±1.28	31.19±1.11	22.65±8.81	0.608	0.000*
*t’*	10.320	11.372	6.514	5.015	12.995	3.167	4.427	12.859		
*P*	0.000*	0.000*	0.000*	0.000*	0.000*	0.000*	0.000*	0.000*		

Note: *P < 0.05 was here considered statistically significant.

**Fig 3:**
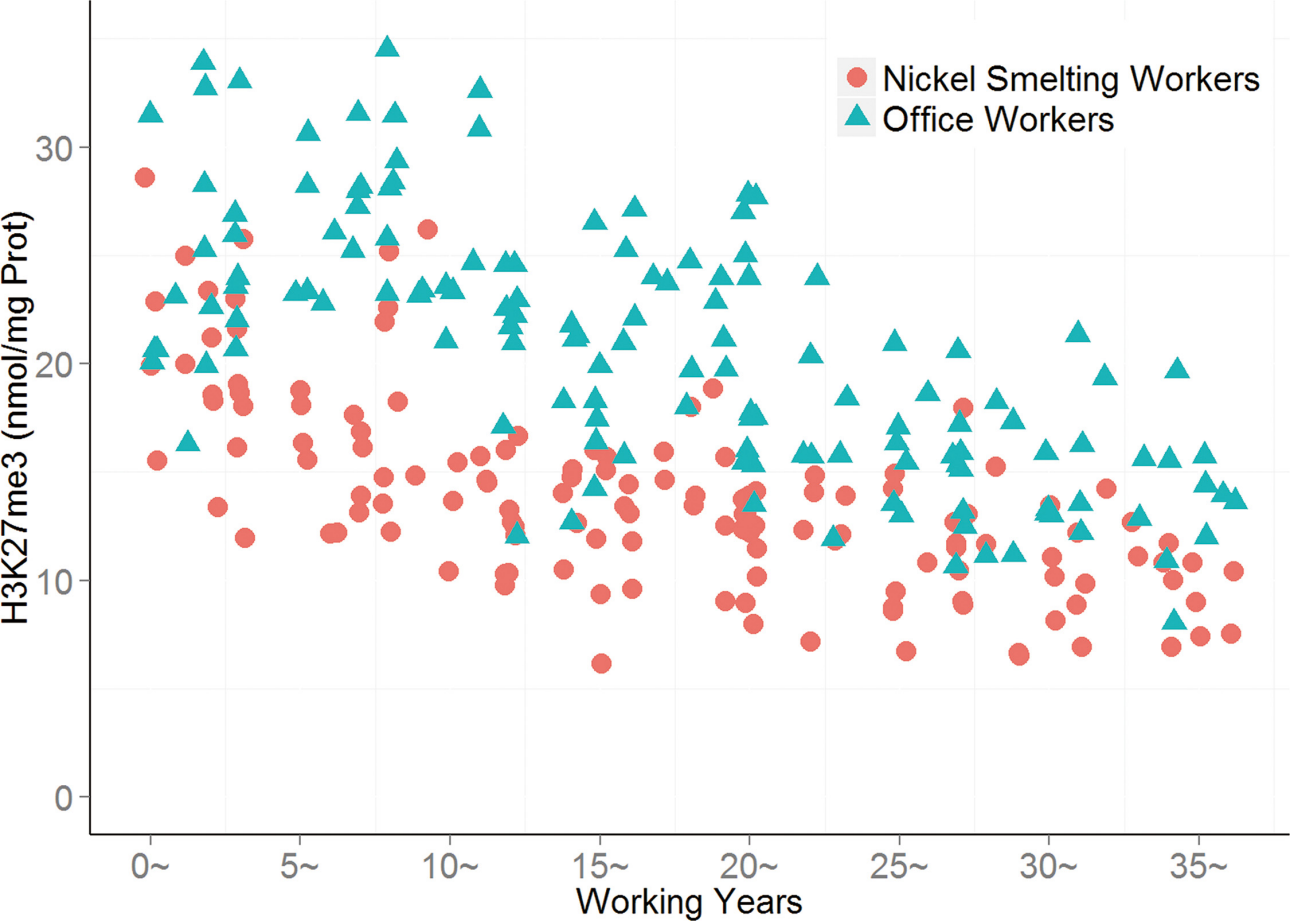
H3K27me3 in smelting and office workers in different working years. The level of H3k27me3 in every experimental subject, the red balls express the smelting workers, and green triangles express the office workers.

As illustrated in Fig 2, in the first service length subgroup (< 5 years), the level of H3K27me3 of nickel-smelting workers was comparable to that of office workers (Fig 3). Starting from the 10–14.99 service length subgroup, the levels of H3K27me3 of nickel-smelting workers were notably lower in the scatter plot and were distinctly different from those of office workers (Fig 3).

After natural logarithm conversion, the H3K27me3 data were found to fit normal distribution. A homogeneity of variance test for different length of service subgroups in the high exposure group showed F = 1.121 and *P* = 0.353 > 0.05, and a test of the control group showed F = 1.234 and *P* = 0.293 > 0.05. In this way, in both cases, there was homogeneity of variance.

As shown in Table 3, In each length of service subgroup, the level of H3K27me3 of nickel-smelting workers was significantly different from that of office workers from the corresponding subgroup (*P* < 0.05), and the level of H3K27me3 of office workers was higher than that of nickel-smelting workers.

**Table 3:** H3K27me3 in nickel smelting and office workers with different lengths of service (nmol/mg prot).

Groups	working years (20 persons in each group)									
	0–4.99	5–9.99	10–14.99	15–19.99	20–24.99	25–19.99	30+	Total	r_s_	*P*
Smelting	19.56±1.24	16.58±1.26	13.09±1.18	13.04±1.31	11.99±1.22	10.67±1.33	9.96±1.24	13.88±4.43	-0.684	0.000*
Office	24.77±1.00	26.90±1.13	21.45±1.27	20.78±1.20	18.63±1.28	15.17±1.22	14.20±1.24	20.67±85.96	-0.709	0.000*
*t*	12.144	67.485	57.568	41.834	38.437	20.483	26.879	-10.813		
*P*	0.001*	0.000*	0.000*	0.000*	0.000*	0.000*	0.000*	0.000*		

Note: *P <0.05, statistically significant.

## Discussion

Previous epidemiological investigations on human subjects and experimental results from cell lines and animal models have indicated that nickel and nickel-containing compounds are mutagens and carcinogens[13–15]. However, the exact molecular mechanism underlying nickel carcinogenicity remains unclear.

**Fig 4:**
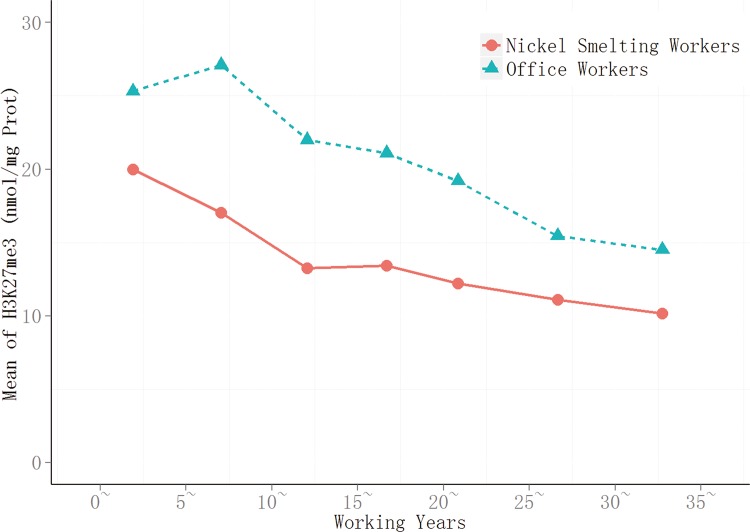
Trend of H3K27me3 with increasing working years. The trend of H3k27me3 in two group with incressing working years, the line connected with red balls express the smelting workers, and with green triangles express the office workers.

Given the low mutagenic capacity of nickel and its compounds, epigenetic changes may offer an alternative explanation for nickel-associated cancer development and provide pre-clinical biomarkers[16]. In the present cohort study, global changes in histone modification were investigated in response to nickel exposure using peripheral blood cells from nickel-smelting and office workers in nickel-smelting factory. Length of service was found to be associated with increased levels of H3K4me3 (Figs 1 and 2) in both nickel-smelting and office workers, this may be taken as an effect of different levels of exposure to nicke, or partly an effect of age. The effect of nickel exposure is was supported by recent cohort study using peripheral blood mononuclear cells from nickel refinery workers[9]. The global level of H3K27me3 was found to be significantly decreased in response to nickel exposure overtime (Figs 3 and 4). The level of H3K4me3 was found to be positively associated with the duration of nickel exposure (Figs 1 and 2), but the level of H3K27me3 was negatively associated with the duration of nickel exposure (Figs 3 and 4) for both nickel-smelting and office workers. Results showed that the levels of H3K4me3 and H3K27me3 were significantly different between nickel-smelting and office workers exposed for the same length of time (Figs 1 and 2), suggesting a dosage effect on those histone modifications. These results suggest that the global level of H3K4me3 and H3K27me3 would provide one of potential biomarkers for nickel exposure in a manner dependent on both length of exposure and dosage.

Epigenetics has been shown to play a critical role in cancer development by changing the state of chromatin, activating oncogenes, and inhibiting the expression of tumor suppressor genes[17]. H3K4me3 has been shown to be associated with gene activation, while H3K27me3 has been shown to be associated with gene repression[18,19]. Deregulation of both histone methylation and the mutation of their regulatory enzymes, such as Mll family members for H3K4 methylation and polycomb repressive complexes (PRC) for H3K27 methylation, has been observed in the development of various cancers[20,21]. Probably due to the weak mutagenicity of nickel and its compounds epigenetic changes have received increasing scrutiny in nickel carcinogenesis studies, but little emphasis was placed on histone acetylation, methylation ubiquitination, DNA methylation, or the regulation of transcription factors[22–24]. Nickel exposure has been shown to be associated with decreased histone acetylation and increased H3K4me3 mark globally[9]. Functionally, both the phenotype and gene expression profile of nickel-transformed cells were reversed to that of untransformed cells by treatment with HDAC inhibitor trichostatin A (TSA), suggesting that nickel exposure might lead to the inhibition of histone acetylation during transformation[25]. Mechanistically, Ni^2+^ has been shown to compete with iron *and with* a co-factor of Jumonji-domain-containing histone demethylase (JMJD) family members *in vitro*. This inhibits KDM3A/JMJD1A activity and results in the increased H3K9 methylation upon exposure to nickel or its compounds[26]. It is here speculated that nickel exposure has similar effect on H3K4 demethylase, such as KDM5A/JARID1A, to repress its activity towards H3K4me3 and lead to the increased H3K4me3. In addition, nickel is a hypoxia mimetic and H3K4me3 has been shown to be increased in hypoxia environment through inhibiting the activity of H3K4 demethylase KDM5A/JARID1A[27]. Consistent with experimental results and mechanisms derived through *in vitro* assays, the first cohort study recently showed the increased H3K4me3 in human blood cells from nickel finery workers. This was here replicated using human blood cells from subjects in the nickel-smelting industry. New biomarkers for global decreased H3K27me3 should be identified. Furthermore, current results suggested the change of global level of H3K4me3 and H3K27me3 to be possibly associated with both length and dosage of nickel exposure. In line with these findings, exposure to Cr^6+^, a mutagen and carcinogen similar to Ni^2+^, has been shown to be associated with increased incidence of lung cancer[28], and with the elevation of H3K4me3 and reduction of H3K27me3 in human A549 cell line[29], suggesting similar histone modification changes in response to metal exposure may promote cancer development.

In summary, the current results showed the global increased H3K4me3 and decreased H3K27me3 to be associated with nickel exposure. Given that nickel exposure is associated with increased lung cancer, it is here speculated that aberrant H3K4me3 and H3K27me3 may change chromatin state more similar to that of cancer cells and therefore facilitate cancer development. Current findings suggest the change of histone methylation may provide one of potential biomarkers for early prediction and evaluation of nickel exposure-associated disease development. Further studies are expected to provide more insight into the mechanisms by which nickel exposure leads to lung cancer development for cancer prevention, diagnostics, and therapeutics. We had limited data on the different levels of nickel exposure from either measuring the levels of nickel in working places or the urinary nickel levels from subjects. Because of the lack of detailed information on nickel exposure, the study cannot establish a correlation between nickel air levels or urinary nickel levels and the changes in levels of histone modifications. Further, we have started collecting the first follow-up data for the “Jinchang cohort”[30], the urine samples have been collected and we are currently processing the samples for urinary nickel testing. Finally we have been trying our best to obtain the data regarding the levels of nickel in the air of various plants.

## Supporting Information

S1 Dataset The dataset is the OD of H3K27me3 and H3K4me3 by ELISA. And the group 1 express the smelting workers, the group 2 express the officers workers.
(XLSX)

## References

[1] Davis JR. ASM Specialty Handbook: Nickel, Cobalt, and Their Alloys. ASM International; 2000.

[2] Cempel M, Nikel G. Nickel: A review of its sources and environmenal toxicology. Polish J Environ Stud. 2006;15(3): 375–382.

[3] Doll R, Mathews JD, Morgan LG. Cancers of the lung and nasal sinuses in nickel workers: a reassessment of the period of risk. Br J Ind Med.1977; 34(2):102–105. 871439 10.1136/oem.34.2.102 PMC1008188

[4] Kasprzak KS, Sunderman FW Jr, Salnikow K. Nickel carcinogenesis. Mutation Research. 2003;533(1–2)533:67–97. 14643413 10.1016/j.mrfmmm.2003.08.021

[5] Ma L, Bai YN, Pu HQ, He J, Bassing BA, Dai M, et al A Retrospective Cohort Mortality Study in Jinchang, the Largest Nickel Production Enterprise in China. Biomed Environ Sci. 2014;27:567–571. 10.3967/bes2014.088 25073918

[6] She J, Yang P, Hong Q, Bai C. Lung Cancer in China:Challenges and Interventions. Chest.2013:143(4):1117–1126. 10.1378/chest.11-2948 23546484

[7] Chervona Y, Arita A, Costa M. Carcinogenic Metals and the Epigenome: Understanding the effect of Nickel, Arsenic, and Chromium.Metallomics.2012;4:619–627. 10.1039/c2mt20033c 22473328 PMC3687545

[8] Sewack GF, Elis TW, Hansen U. Binding OF TATA binding protein to a naturally positioned nucleoaome is facilitated by histone acetylation. Mol Cell Biol.2001:21(4):1404–1415. 11158325 10.1128/MCB.21.4.1404-1415.2001 PMC99592

[9] Arita A, Niu JP, Qu QS, Zhao NJ, Ruan Y, Nadas A, et al Global levels of histone modifications in peripheral blood mononuclear cells of subjects with exposure to nickel.Environ Health Perspect.2012;120(2):198–203. 10.1289/ehp.1104140 22024396 PMC3279455

[10] Hattori N, Ushijima T. Comendium of aberrant DNA methylation and histone modifications in cancer.Biochem Biophys Res Commun 9 4 2014 10.1016/j.bbrc.2014.08.140 25194808

[11] Chen H, Ke Q, Kluz T, Yan Y, Costa M. Nickel ions increase histone H3 lysine 9 dimethylation and induce transgene silencing. Mol Cell Biol. 2006;26:3728–3737. 16648469 10.1128/MCB.26.10.3728-3737.2006 PMC1488989

[12] Zhou X, Li Q, Arita A, Sun H, Coata M. Effects of nickel, chromate, and arsenite on histone 3 lysine methylation. Toxicol Appl Pharmacol.2009;236(1):78–84. 10.1016/j.taap.2009.01.009 19371620 PMC2684878

[13] Govindarajan B, Klafter R, Miller MS, Mansur C, Mizesko M, Bai XH, et al Reactive oxygen-induced carcinogenesis causes hypermethylation of p16(Ink4a) and activation of MAP kinase. Mol Med.(2002;8(1):1–8. 10.1016/j.ejvs.2004.01.021 11984000 PMC2039931

[14] Yang LQ, Ji WD, Tao GH, Zhang WJ, Gong CM, Zhou L, et al Genome DNA hypomethylation in the process of crystalline nickel-induced cell malignant transformation. Zhonghua Yu Fang Yi Xue Za Zhi. 2010; 44(7):622–625. 21055078

[15] Report of the International Committee on Nickel Carcinogenesis in Man. Scand J Work Environ Health. 1990; 16:1–82. 10.5271/sjweh.1813 2185539

[16] Suu H, Shamy M, Costa M. Nickel and epigenetic gene silencing.Gene.s. 2013;4:583–595. 10.3390/genes4040583 PMC3927569 24705264

[17] Barrow TM, Michels KB. Epigenetic epidemiology of cancer. Biochem Biophys Res Communications. 2014;1–14. 10.1016/j.bbrc.2014.08.002 25124661

[18] Sawan C, Herceg Z. Histone modifications and cancer. Adv Genet. 2010; 70:57–85. 10.1016/S0065-Z660(10)70003-1 20920745

[19] Ursula MN, John MS. Epigenetic Control of Aging. Antioxidants & redox signaling 2011;14(2):241–259. 10.1089/ars.2010.3250 PMC3014766 20518699

[20] Wang PF, Lin CQ, Edwin RS, Guo H, Brian WS, Wu M, et al Global Analysis of H3K4 Methylation defines MLL Family Member Targets and Points to a Role for MLL1-Mediated H3K4 Methylation in the Regulation of Transcriptional Initiation by RNA Polymerase II. Mol Cell Biol.2009; 29(22):6074–6085. 10.1128/MCB.00924-09 19703992 PMC2772563

[21] Yap DB, Chu J, Berg T, Schapira M, Cheng SW, Moradian A, et al Somatic mutations at EZH2 Y641 act dominantly through a mechanism of selectively altered PRC2 catalytic activity, to increase H3K27 trimethylation. Blood. 2011;117(8):2451–2459. 10.1182/blood-2010-11-321208 21190999 PMC3062411

[22] Ke Q, Davidson T, Chen H, Kluz T, Costa M. Alterations of histone modifications and transgene silencing by nickel chloride. Carcinogenesis2.2006;27(7):1481–1488. 10.1093/carcin/bg1004 16522665

[23] Lee YW, Klein CB, Kargacin B. Carcinogenic nickel silences gene expression by chromatin condensation and DNA methylation: a new model for epigenetic carcinogens. Mol Cell Biol.1995;15:2547–2557. 7537850 10.1128/mcb.15.5.2547 PMC230485

[24] Kowara R, Salnikow K, Diwan BA, Bare RM, Waalkes MP, Kasprzak KS. Reduced Fhit protein expression in nickel-transformed mouse cells and in nickel-induced murine sarcomas. Mol Cell Biochem.2004;255(1–2):195–202. 14971660 10.1023/b:mcbi.0000007275.22785.91

[25] Yan Y, Kluz T, Zhang P, Chen Hb, Costa M. Analysis of specific lysine histone H3 and H4 acetylation and methylation status in clones of cells with a gene silenced by nickel exposure. Toxicology and Applied Pharmacology. 2003;190:272–277. 10.1016/S0041-008x(03)00169-8 12902198

[26] Chen H, Kluz T, Zhang RH, Costa M. Hypoxia and nickel inhibit histone demethylase JMJD1A and repress Spry2 expression in human bronchial epithelial BEAS-2B cells. Carcinogenesis. 2010;31(12):2136–2144. 10.1093/carcin/bgq197 20881000 PMC2994281

[27] Zhou X, Sun H, Chen HB, Zavadil J, Kluz A, Arita A, et al Hypoxia induces trimethylated H3 lysine 4 by inhibition of JARID1A demethylase.Cancer Res. 2010;70:4214–4221. 10.1158/0008-5472.CAN-09.2942 20406991 PMC3007597

[28] Katz AJ, Chiu A, Beaubier J, Shi X. Combining Drosophila melanogaster somatic-mutation- recombination and electron-spin-resonance-spectroscopy data to interpret epidemiologic observations on chromium carcinogenicity. Mol Cell Biochem.2001;222:61–68. 10.1007/978-1-4615-0793-2-8 11678612

[29] Sun H, Zhou X, Chen H, Li Q, Costa M. 237:258–266. Modulation of histone methylation and MLH1 gene silencing by hexavalent chromium. Toxicol Appl Pharmacol.2009;237(3):258–266. 10.1016/j.taap.2009.04.008 19376149 PMC2701251

[30] Bai YN, Yang AM, Pu HQ, He J, Zheng TZ, Cheng N, et al Nickel-exposed workers in China: a cohort study. Biomed Environ Sci. 2014;27(3):208–211. 10.3967/bes2014.042 24709102

